# Antimicrobial Activity and Composition of Five *Rosmarinus* (Now *Salvia* spp. and Varieties) Essential Oils

**DOI:** 10.3390/antibiotics10091090

**Published:** 2021-09-09

**Authors:** Ylenia Pieracci, Daniela Ciccarelli, Silvia Giovanelli, Luisa Pistelli, Guido Flamini, Claudio Cervelli, Francesca Mancianti, Simona Nardoni, Fabrizio Bertelloni, Valentina Virginia Ebani

**Affiliations:** 1 Department of Pharmacy, University of Pisa, 56121 Pisa, Italy; yleniapieracci@gmail.com (Y.P.); silvia.giovanelli84@gmail.com (S.G.); luisa.pistelli@unipi.it (L.P.); 2 Department of Biology, University of Pisa, 56126 Pisa, Italy; daniela.ciccarelli@unipi.it; 3 Interdepartmental Research Center “Nutraceuticals and Food for Health” (NUTRAFOOD), University of Pisa, 56121 Pisa, Italy; francesca.mancianti@unipi.it; 4 CREA (Consiglio per la Ricerca in Agricoltura e l’Analisi dell’Economia Agraria)—Research Center for Vegetables and Ornamental Crops, 18038 Sanremo, Italy; claudio.cervelli@crea.gov.it; 5 Department of Veterinary Sciences, University of Pisa, 56124 Pisa, Italy; simona.nardoni@unipi.it (S.N.); fabrizio.bertelloni@unipi.it (F.B.)

**Keywords:** *Rosmarinus officinalis*, *Rosmarinus eriocalyx*, ‘Boule’, ‘Vicomte de Noailles’, ‘Gorizia’, ‘Joyce de Baggio’, hydrodistillation, agar diffusion test, MIC, Principal Component Analysis (PCA)

## Abstract

*Salvia rosmarinus* Spenn. and *Salvia jordanii* J.B.Walker are aromatic evergreen shrubs belonging to the Lamiaceae family. Their aerial parts have been used since ancient times as natural preservatives. The present study reported the investigation of the chemical profile and the extraction yield of the essential oils (EOs) obtained from the dried aerial parts of four cultivars of *Salvia rosmarinus* (‘Boule’; ‘Vicomte de Noailles’; ‘Gorizia’; ‘Joyce de Baggio’) and the species *S. jordanii*, together with their antibacterial and antifungal activities. The phytochemical investigation evidenced a predominance of oxygenated monoterpenes in all the samples (57.5–77.1%), except in ‘Boule’, in which the hydrocarbon form prevailed (50.2%). Principal Component Analysis (PCA) of the matrix taxa × compounds showed that nine compounds have a significant discriminating function between the samples. ‘Vicomte de Noailles’ was characterized by high amounts of camphor and 14-hydroxy-9-*epi-(E)-*caryophyllene, while ‘Gorizia’ and Jord differed in their predominance of camphene, borneol, bornyl acetate, and α-humulene. Lastly, ‘Boule’ and ‘Joyce de Baggio’ segregated separately and were characterized by high amounts of α-pinene, myrcene, and verbenone. The selected EOs presented a moderate antibacterial activity on the tested bacterial strains and resulted not active on the tested yeast species.

## 1. Introduction

The genus *Rosmarinus* L., belonging to the Lamiaceae family, was recently included in the genus *Salvia* L. [1]. *Salvia* subg. *rosmarinus* comprise three species of aromatic plants [2]: *Salvia rosmarinus* Spenn. (synonym of *Rosmarinus officinalis* L., and isonym of *Salvia rosmarinus* Schleid), *Salvia jordanii* J.B.Walker (synonym of *Rosmarinus eriocalyx* Jord. & Fourr.), and *Salvia granatensis* B.T.Drew (synonym of *Rosmarinus tomentosus* Hub.-Mor. & Maire) [3,4]; only the first two species are widely used in traditional medicine [5] and as cooking ingredients [6].

*S. rosmarinus*, commonly known as “rosemary”, is the most known species. It is an evergreen shrub, able to grow in every type of soil, with predilection for dry and rocky ones. It is no coincidence that it is widespread in the Mediterranean area, in particular in the coastal scrub [7]. It is widely used for its aromatic and medicinal properties [8], determined by its content in secondary metabolites. Among these, the essential oil (EO) responsible for the pleasant smell is the principal product obtained from this plant by industries [9], as it can be exploited for the treatment of skin, digestive, and renal problems, as well as for headaches [10]. On the basis of its main EO components, *S. rosmarinus* can be distinguished in different major chemotypes, including *cineoliferous* (1,8-cineole > 40%) [2], *camphoriferous* (camphor > 20%), *verbenoniferoum* (verbenone > 15%) [2,11], and *α-pinene chemotype* (α-pinene as the major component) [9]. The chemical composition of rosemary essential oil has been widely investigated in relation to different geographical locations of collection, environmental conditions, and seasonal periods. As reported by Cioni et al., soil and climatic conditions only partially modulate the biosynthesis of the main chemicals of the EO which, instead, is mainly determined by the genetic heritage of the plant [12]. Nevertheless, the differences are mainly quantitative rather than qualitative [5].

*S. jordanii* (ex. *R. eriocalyx* Jord. & Fourr., previously known as *R. tournefortii* De Noé), is an aromatic evergreen bush like rosemary, but it has been introduced into cultivation only in the last decades [13]. It is typical of Algeria, Spain and Morocco, preferring mountain rocky grounds and pastures. It differs from *S. rosmarinus* for some morphological characters such as the smaller leaves, the woolly calyx, and the prostrate growth [6]. As opposite to *S. rosmarinus*, moreover, only a few studies have been conducted on the chemical composition of *S. jordanii* essential oil, most of which also investigated its antibiotic activity [5,13,14,15].

The essential oils obtained from both *S. rosmarinus* and *S. jordanii* have been reported in numerous studies for their antibacterial and antifungal properties, as the aerial part of these plants have been widely used since ancient times as natural preservatives [9,16]. Abers et al. reported the essential oil of *R. officinalis* as a good broad-spectrum antibacterial agent [17], and Soulaimani et al. highlighted a better activity against Gram positive bacteria than against the Gram negative ones [18]. In recent times, the EOs, which are complex mixtures of secondary metabolites characterized by high volatility and strong smell [19], have received significant interest for their antibacterial and antifungal properties given the change in consumer behaviour toward a preference for natural products [16,20].

The aim of the present work was to investigate the chemical composition and the extraction yield of the EOs obtained from the dried flower tops of four cultivars of *S. rosmarinus* (‘Boule’; ‘Vicomte de Noailles’; ‘Gorizia’; ‘Joyce de Baggio’) and one species of *S. jordanii*, all cultivated in the same geographical area (Sanremo, Italy) and with identical growing conditions, together with their antibacterial and antifungal properties.

## 2. Results and Discussions

### 2.1. Phytochemical Investigation

The complete compositions and the extraction yields of the essential oils (EOs) obtained from the dried aerial part of the samples are reported in Table 1. The following taxa acronyms were used: Boule = *S. rosmarinus* ‘Boule’, Gori = *S. rosmarinus* ‘Gorizia’, Joyce = *S. rosmarinus* ‘Joyce de Baggio’, Vicom = *S. rosmarinus* ‘Vicomte de Noailles’, and Jord = *S. jordanii.* Overall, 65 compounds were identified, accounting for 98.6–100% of the total composition.

According to Flamini et al. (2020) [2] monoterpenes are the main class of compounds in *Rosmarinus* genus: indeed, the EO obtained from *S. rosmarinus* ‘Boule’ was characterized by a predominance of their hydrocarbon derivatives (50.2%), while those obtained from the other four samples presented more oxygenated ones.

Nevertheless, monoterpene hydrocarbons were well-represented in all the samples, accounting for up to 50.2% in Boule, followed by Joyce (36.0%), Gori (24.0%), Jord, and Vicom (14.3 and 14.2%, respectively). α-Pinene, camphene, β-pinene, and limonene were the main chemicals of this class, but only the first reached considerable relative amounts, up to 37.3% in ‘Boule’ and 25.6% in ‘Joyce’.

Oxygenated monoterpenes, indeed, resulted more abundant in Jord and Vicom (77.1% and 71.9%, respectively), followed by Joyce and Gori (60.9% and 57.5%, respectively) and Boule (46.9%). Within this chemical class, 1,8-cineole (11.4–23.9%), camphor (3.3–42.2%), borneol (0.6–14.6%), 4-terpineol 0.9–2.8%), α-terpineol (2.0–2.9%), verbenone (0.6–14.9%), and bornyl acetate (0.2–10.8%) were the most representative compounds, as they were detected in all the samples, even though with a high variability in their relative abundances.

Sesquiterpenes were also detected in appreciable relative amounts: the EOs obtained from Gori and Jord presented a predominance of the hydrocarbons form, and Vicom of the oxygenated form. In Boule and Joyce, this chemical class was poorly represented. β-Caryophyllene (0.2–6.7%) and caryophyllene oxide (0.4–4.3%) were identified in each sample; noteworthy was the amount of 14-hydroxy-9-*epi-(E)-*caryophyllene in Vicom (7.1%).

All the most representative compounds detected in the EOs were typical chemicals of the essential oils of *S. rosmarinus* [9] and *S. jordanii* [5,14].

The EO extraction yield presented significant differences among the samples: Vicom was the most productive one (2.25% *w/w*), followed by Gori (1.17% *w/w*), while Joyce, Jord, and Boule presented the lowest yields (0.76 > 0.71 > 0.56% *w/w*, respectively).

This is the first time that the essential oil composition of the four cultivars of *S. rosmarinus* (‘Boule’; ‘Vicomte de Noailles’; ‘Gorizia’; ‘Joyce de Baggio’) was reported to better utilise this plant material not only as ornamental display items but also as derivative products for industrial use.

#### Statistical Analysis

**Figure 1:**
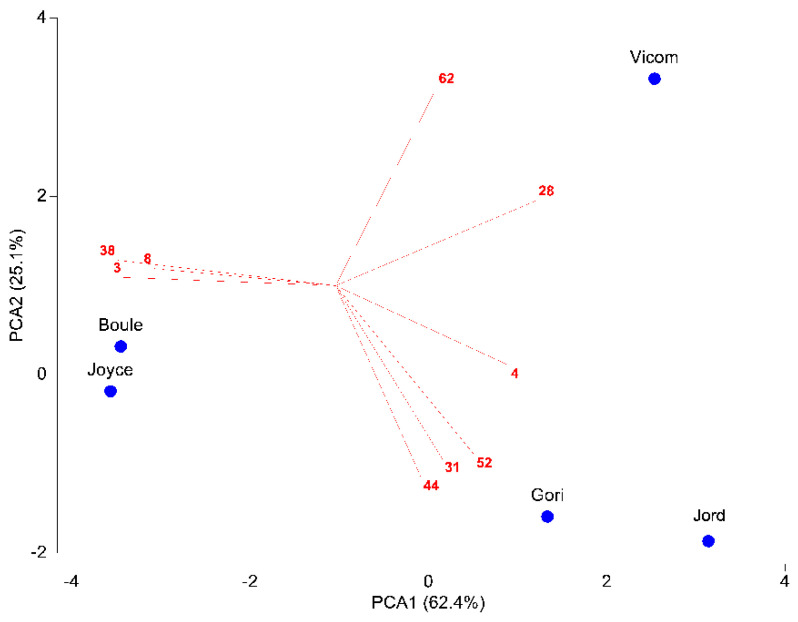
PCA of the matrix 5 taxa × 18 compounds. Compounds with a Pearson correlation coefficient > 0.8 with the first two PCA axes are shown. Abbreviations of chemical compounds: 3 = α-pinene, 4 = camphene, 8 = myrcene, 28 = camphor, 31 = borneol, 38 = verbenone, 44 = bornyl acetate, 52 = α-humulene, 62 = 14-hydroxy-9-*epi-(E)-*caryophyllene.

The first axis of PCA explained 62.4% of variance, the second axis (PCA2) a further 25.1% (Figure 1). Vicom segregated alone, while the other taxa were distributed into two groups, one formed by Gori and Jord, and the other one made up by Boule and Joyce. Nine chemical compounds showed a significant discriminative function between the taxa. Vicom was characterized by high amounts of camphor (28) and 14-hydroxy-9-*epi-(E)-*caryophyllene (62). Gori and Jord differed in their predominance of camphene (4), borneol (31), bornyl acetate (44), and α-humulene (52). Lastly, Boule and Joyce were characterized by high amounts of α-pinene (3), myrcene (8), and verbenone (38).

### 2.2. Antimicrobial Investigation

In vitro antibiotic sensitivity tests detected multi-resistance of the assayed bacterial isolates (Table 2).

Enterococci emerged as the bacterial isolate with resistance to most antibiotics. *Enterococcus* spp. have a strong ability to acquire, express and transfer antimicrobial resistance [21], and our findings are in agreement with other studies reported in the literature [22].

The diameters of the inhibition zone evaluated with the agar diffusion tests of the EOs on the bacterial strains are reported in Table 3. Results showed that the selected EOs had varying degrees of growth inhibition against the tested bacterial strains. No inhibition zone was observed when DMSO was tested as the negative control, whereas chloramphenicol, included as positive control, proved effective against all isolates.

All the EOs were moderately active against *S*. ser. Typhimurium and *Y. enterocolitica* strains, whilst any EOs presented activity on the strain of *E. durans* and *E. faecium.* Furthermore, all the samples inhibited *L. monocytogenes*, with the only exception of that of Boule, which, instead, was active on *E. faecalis*, inhibited also by the EO obtained from Jord.

Results of minimum inhibitory concentration (MIC) were reported in Table 4.

Values ranging from 10% to 1.25% (*v*/*v*) were recorded in relation to the different EOs and the bacterial isolates. No growth inhibition was observed with the negative control, whereas chloramphenicol resulted active against all strains.

The EO obtained from Joyce was the most active, as it presented 1.25% of MIC for *Y. enterocolitica, L. monocytogenes, E. durans*, and *E. faecium.* The EO obtained from Jord also presented a moderate antibacterial activity on the strains of *Y. enterocolitica, L. monocytogenes*, and *E. faecium*, while Vicom on *E. durans* and *E. faecium* strains. On the contrary, the EOs of Boule and Gori were the less active on the tested bacteria.

Considering the bacterial strain, *S.* ser. Thyphimurium was the most resistant to the tested EOs, in contrast to Jordan et al. (2013) who reported a strong activity of all *S. rosmarinus* chemotypes against this strain. Moreover, they reported a strong activity also against *L. monocytogenes* [23], which in the present study was inhibited only by the EO obtained from Joyce.

On the contrary, *E. durans* proved to be the most susceptible bacterial strain, but no studies are reported in the literature on this matter.

The differences resulting from the agar diffusion tests and the MIC, mainly with enterococci, are remarkable. These findings corroborate the observations previously reported by other authors, which affirmed that diffusion assays are unsuitable to EOs testing because the oil components are partitioned through the agar according to their affinity with water [24,25].

### 2.3. Antimycotic Activity

The anti-yeast activity of the EOs is reported in Table 5. The EOs resulted not active on the tested yeast species. Results of the conventional drugs assay was consistent with data from the literature [26,27]. *C. guilliermondii* and *S. cerevisiae* scored sensitive to all selected drugs; *C. albicans* and *C. tropicalis* were resistant to fluconazole and anidulafungin, respectively; *C. krusei* scored resistant to caspofungin and fluconazole; and *C. parapsilosis* was resistant to anidulafungin, caspofungin and fluconazole.

Concerning the *S. rosmarinus* EO, the findings of the present study are in alignment with those of Satyal et al. [8], while in the case of *S. jordanii* they are in contrast with those of Maqbul et al. (2020), who reported good antifungal activity against *C. albicans* [15].

To the best of our knowledge, this is the first study that evaluated the antimicrobial activity of *S. jordanii* and different varieties of *S. rosmarinus* against bacterial and *Candida* isolates cultured from poultry clinical cases.

## 3. Materials and Methods

### 3.1. Plant Material

The plant material, reported in Table 6, belonged to the collection coming from CREA- Sanremo, Centro di Ricerca Orticoltura e Florovivaismo, located in Sanremo, Italy (43°49’ N, 07°45’ E). The rooting of rosemary varieties was carried out in a greenhouse under a small semicircular top section tunnel about 60 cm tall with a mesh metal structure welded and overcoated with a 70% black shading net. The rooting substrate was composed of a mixture of 50% Klassman Traysubstrat and agriperlite (3 mm). The cuttings were a half-woody type with a length ranging between 5 and 8 cm depending on the variety; they have been cleared of leaves for about 1.5 cm in the lower portions before insertion into the substrate. A commercial growth regulator for rooting was used (Germon—Gobbi- for woody cuttings, powder) containing naphthalene acetic acid (NAA) 0.75%. Containers for cuttings consisted of 60-hole alveolar panels (Florpack), with a hole diameter of 4.2 cm. Irrigation during the rooting was manually performed by rain system 1–2 times per day. The rooting period started at the beginning of September and ended in late October, with a high percentage of rooting (70–90%). For rosemary, a substrate for nursery plants (Terflor Vulcan) was used. The plants were grown in an open field. The irrigations were performed with a timed automatic system, with frequency depending on the season (2–3 times weekly watering in winter mainly on small plants, daily in summer). Nutrients were given by a 1.5 g/L solution containing N: P_2_O_5_: K_2_O: MgO (15: 10: 15: 2 + MgO), plus microelements.

### 3.2. Phytochemical Investigation

#### 3.2.1. Essential oil (EO) Hydrodistillation

The essential oils were obtained from the dried flowering tops of the plant by means of hydrodistillation performed with a standard *Clevenger*-type apparatus for 2 h. For all the samples, the hydrodistillation was accomplished in triplicate on 50 g of plant material and the collected essential oils were diluted to 0.5% in HPLC-grade *n*-hexane before the injection in the GC–MS apparatus.

#### 3.2.2. Gas Chromatography–Mass Spectrometry Analyses

For Gas Chromatography/Electron Ionization Mass Spectrometry (GC/EI-MS), an Agilent 7890B gas chromatograph (Agilent Technologies Inc., Santa Clara, CA, USA) equipped with an Agilent HP-5MS capillary column (30 m × 0.25 mm; coating thickness 0.25μm) and an Agilent 5977B single quadrupole mass detector was used. The analytical conditions were as follows: the oven temperature was programmed to rise from 60 °C to 240 °C at 3 °C/min; the injector temperature was 220 °C; the transfer-line temperature was 240 °C; the carrier gas was He (1 mL/min). The acquisition parameters were as follows: full scan; scan range: 35–300 *m*/*z*; scan time: 1.0 sec; threshold: 1 count. The identification of the constituents was based on the comparison of their retention times (tR) with the retention time of pure reference samples, comparing their linear retention indices (LRIs) relative to the series of *n*-alkanes. The mass spectra were compared with those listed in the commercial libraries NIST 14 and ADAMS and in a home-made mass-spectral library, built using MS literature [28,29] combined with data experimentally obtained from pure substances and commercial essential oils of known composition.

#### 3.2.3. Statistical Analysis

The analysis of variance (ANOVA) was carried out on the classes of compounds and on the EO extraction yield using the JMP software package (SAS Institute, Cary, NC, USA). Averages were separated by Tukey’s b post hoc test. *p* < 0.05 was used to assess the significance of differences between means.

All data from the different cultivars were merged in one matrix taxa × chemical compounds (values under 1.5% were excluded for the purpose of statistical analysis). We performed a Principal Component Analysis (PCA) using a square root transformed matrix (5 taxa per 18 compounds). The Pearson correlation has been calculated to analyze the effects of chemical compounds on taxa. The statistical multivariate analyses were done with PRIMER v.7 software (PRIMER-E, Plymouth [30]).

### 3.3. Antimicrobial Investigation

#### 3.3.1. Antibacterial Activity

##### Bacterial Strains

EOs were individually tested against 6 wild bacterial strains belonging to the species *Salmonella enterica* serovar Typhimurium, *Yersinia enterocolitica*, *Listeria monocytogenes*, *Enterococcus durans*, *E. faecalis*, and *E. faecium*. The strains have been previously isolated from poultry fecal samples, typed and stored at −80 °C in glycerol broth. The in vitro antibiotic sensitivity was determined by the Kirby-Bauer agar disc diffusion method [21]. Each isolate was assayed with the following antibiotics (Oxoid): tetracycline (30 µg), ceftazidime (30 µg), rifampicin (30 µg), cephalexin (30 µg), and cefotaxime (30 µg), chloramphenicol. The results were interpreted on the basis of the indications suggested by the National Committee for Clinical Laboratory Standards (NCCLS) [22].

##### Agar Disc Diffusion Method

The Kirby-Bauer agar disc diffusion method was used to determine the antibacterial activity of the EOs following the procedures described by Clinical and Laboratory Standards [31].

A paper disk impregnated with 10 µL of DMSO was included as a negative control, whereas a commercial disk impregnated with chloramphenicol (30 µg) (Oxoid Ltd.) was used as a positive control. Growth inhibition zones were calculated after incubation at 37 °C for 24 h. All tests were performed in triplicate. The results were interpreted on the basis of the indications suggested by the NCCLS [32].

##### Minimum Inhibitory Concentration (MIC)

MIC was tested with the broth microdilution method on the basis of the guidelines of CLSI (1990) [33] and the protocol previously described by Ebani et al. 2016 [34]. The same assay was performed simultaneously for growth control of microorganisms (tested bacterial strains and media) and for sterility control (tested oil and media). Positive control using chloramphenicol (Oxoid) was also included. All tests were performed in triplicate.

#### 3.3.2. Antimycotic Activity

##### Yeasts Species

The efficacy of the selected EOs was tested against 5 *Candida* spp. isolates (*C. albicans*, *C. tropicalis*, *C. guilliermondii*, *C. krusei* and *C. parapsilosis*). *Saccharomyces cerevisiae* can be administered to broilers as a probiotic for its activity on performance and immune modulatory functions [35]. For this reason, the N. 1 isolate of this fungal species was tested to evaluate a possible inhibitory activity of selected EOs. All yeasts had been isolated from poultry droppings and identified by their morphological and physiological features. Definitive identification was achieved by ID32C galleries (BioMerieux, Marcy l’Etoile, France). Fungal strains were stored in distilled water at room temperature until the testing.

##### Microdilution Test

The antimycotic activity of selected EOs was assessed by the broth microdilution method in malt extract broth following the guidelines of EUCAST as modified by Budzynska et al. [36] using sweet almond fatty oil (*Prunus dulcis* Mill. D.A. Webb.) instead of Tween 20 for preparing the yeast suspension. EOs in almond oil were dissolved into the medium and assayed at dilutions (*v*/*v*%) of 10%, 7.5%, 5%, and 2.5%. All tests were carried out in triplicate. Negative and positive controls were achieved. The drug sensitivity pattern of yeasts was checked by Etest (BioMerieux, Marcy l’Etoile, France). Testing was performed as recommended by the manufacturer. Strips containing anidulafungin, amphotericin B, caspofungin, micafungin, fluconazole, posaconazole and voriconazole were used.

## 4. Conclusions

All the analysed EOs were characterized by a predominance of oxygenated monoterpenes (57.5–77.1%), except that of ‘Boule’, in which the hydrocarbon form prevailed (50.2%). Considering the whole chemical composition, a total of nine compounds showed a significant discriminative function between the samples. ‘Vicomte de Noailles’ was characterized by high amounts of camphor and 14-hydroxy-9-*epi-(E)-*caryophyllene; ‘Gorizia’ and Jord differed for their predominance of camphene, borneol, bornyl acetate, and α-humulene and, lastly, ‘Boule’ and ‘Joyce de Baggio’ were characterized by high amounts of α-pinene, myrcene, and verbenone.

Joyce EO was the most active on the tested bacterial strains. All the tested bacterial isolates were previously obtained from poultry clinical cases. The obtained results suggest that the investigated EOs, and primarily the Joyce EO, could be promising natural products to be used for the hygiene of poultry farms. Concerning antimycotic activity, the EOs resulted not active on the tested yeast species. More studies would be advisable to evaluate the antibacterial activity of different mixtures of the most active tested EOs.

## Figures and Tables

**Table 1:** Complete composition and extraction yield (% *w/w* dry weight) of the essential oil obtained from the samples of *S. rosmarinus* and *S. jordanii*.

Peak	Compounds	l.r.i.	Class.	Relative Abundances (%) ±SD				
				Boule	Gori	Joyce	Vicom	Jord
**1**	**tricyclene**	**922**	**mh**	**-**	**0.1 ± 0.00**	**-**	**-**	**0.1 ± 0.01**
2	α-thujene	926	mh	-	0.2 ± 0.02	0.1 ± 0.00	0.2 ± 0.02	-
**3**	**α-pinene**	**933**	**mh**	**37.3 ± 3.09**	**6.4 ± 0.10**	**25.6 ± 0.02**	**4.1 ± 0.04**	**3.1 ± 0.17**
**4**	**camphene**	**948**	**mh**	**2.9 ± 0.09**	**4.3 ± 0.04**	**2.0 ± 0.09**	**3.3 ± 0.10**	**3.9 ± 0.06**
5	thuja-2,4(10)-diene	954	mh	0.3 ± 0.02	-	0.4 ± 0.07	-	-
6	β-pinene	977	mh	0.5 ± 0.04	4.2 ± 0.40	1.8 ± 0.07	1.6 ± 0.03	0.9 ± 0.01
7	3-octanone	985	nt	-	1.0 ± 0.17	-	-	-
**8**	**myrcene**	**991**	**mh**	**2.0 ± 0.16**	**0.6 ± 0.01**	**0.9 ± 0.09**	**0.4 ± 0.03**	**0.2 ± 0.02**
9	α-phellandrene	1006	mh	-	1.2 ± 0.05	0.2 ± 0.00	-	-
10	δ-3-carene	1011	mh	-	0.4 ± 0.01	-	-	-
11	α-terpinene	1017	mh	0.2 ± 0.01	0.5 ± 0.02	0.5 ± 0.01	0.3 ± 0.01	0.7 ± 0.01
12	*p*-cymene	1025	mh	2.8 ± 0.22	1.0 ± 0.23	0.8 ± 0.08	1.8 ± 0.09	1.6 ± 0.06
13	limonene	1029	mh	3.3 ± 0.11	3.6 ± 0.05	2.1 ± 0.12	1.7 ± 0.14	1.7 ± 0.03
14	1,8-cineole	1031	om	11.4 ± 0.22	20.5 ± 0.73	23.9 ± 0.42	20.0 ± 0.64	11.5 ± 0.11
15	*(Z)-*β-ocimene	1036	om	-	-	-	-	1.2 ± 0.10
16	γ-terpinene	1058	mh	0.4 ± 0.03	1.0 ± 0.10	0.9 ± 0.02	0.6 ± 0.02	0.7 ± 0.02
17	*cis*-sabinene hydrate	1066	om	-	0.3 ± 0.02	-	0.1 ± 0.01	0.1 ± 0.01
18	terpinolene	1089	mh	0.5 ± 0.02	0.6 ± 0.01	0.7 ± 0.03	0.4 ± 0.00	0.2 ± 0.01
19	*trans*-sabinene hydrate	1098	om	-	0.1 ± 0.02	-	-	-
20	linalool	1101	om	1.5 ± 0.01	0.4 ± 0.04	2.1 ± 0.14	0.2 ± 0.01	-
21	filifolone	1108	om	0.2 ± 0.00	-	-	-	-
22	fenchol	1114	om	0.1 ± 0.01	-	-	-	-
23	*cis-p*-menth-2-en-1-ol	1122	om	-	-	0.2 ± 0.03	-	-
24	α-campholenal	1125	om	-	-	-	0.2 ± 0.01	-
25	chrysanthenone	1126	om	0.8 ± 0.05	0.2 ± 0.06	0.2 ± 0.01	-	-
26	*trans*-pinocarveol	1139	om	0.2 ± 0.02	0.1 ± 0.02	-	-	-
27	*cis*-verbenol	1142	om	-	0.1 ± 0.03	0.1 ± 0.03	-	-
**28**	**camphor**	**1145**	**om**	**7.7 ± 0.22**	**16.9 ± 1.35**	**3.3 ± 0.50**	**42.2 ± 0.52**	**33.4 ± 0.38**
29	*trans*-pinocampone	1160	om	0.3 ± 0.01	-	0.2 ± 0.01	0.2 ± 0.02	-
30	pinocarvone	1163	om	0.2 ± 0.00	0.3 ± 0.02	0.3 ± 0.03	0.3 ± 0.07	-
**31**	**borneol**	**1165**	**om**	**2.5 ± 0.11**	**6.5 ± 0.00**	**3.7 ± 0.26**	**0.6 ± 0.13**	**14.6 ± 0.01**
32	*iso*pinocampheol	1173	om	0.4 ± 0.04	-	-	-	-
33	*cis-*pinocamphone	1174	om	-	0.8 ± 0.02	0.8 ± 0.01	0.5 ± 0.00	-
34	4-terpineol	1177	om	1.4 ± 0.08	0.9 ± 0.08	1.1 ± 0.01	1.2 ± 0.02	2.8 ± 0.03
35	*p-*cymen-8-ol	1185	om	0.1 ± 0.01	-	-	0.2 ± 0.01	-
36	α-terpineol	1191	om	2.6 ± 0.19	2.0 ± 0.24	2.4 ± 0.09	2.8 ± 0.07	2.9 ± 0.06
37	myrtenol	1195	om	0.2 ± 0.02	0.2 ± 0.02	0.4 ± 0.14	0.2 ± 0.03	-
**38**	**verbenone**	**1210**	**om**	**12.8 ± 2.67**	**1.9 ± 0.15**	**14.9 ± 0.27**	**2.7 ± 0.03**	**0.6 ± 0.02**
39	*trans*-carveol	1219	om	0.1 ± 0.06	-	-	0.2 ± 0.04	-
40	carvone	1244	om	-	-	-	0.1 ± 0.03	-
41	geraniol	1254	om	0.5 ± 0.08	-	3.9 ± 0.15	-	-
42	*trans*-ascaridol glycol	1268	om	-	0.4 ± 0.10	-	-	-
43	geranial	1271	om	-	-	0.3 ± 0.02	-	-
**44**	**bornyl acetate**	**1286**	**om**	**3.9 ± 0.45**	**6.5 ± 0.50**	**2.6 ± 0.17**	**0.2 ± 0.01**	**10.8 ± 0.06**
45	myrtenyl acetate	1326	om	-	-	0.1 ± 0.00	-	-
46	eugenol	1357	pp	-	-	-	-	0.4 ± 0.02
47	α-copaene	1376	sh	-	0.3 ± 0.05	-	-	-
48	geranyl acetate	1385	om	-	-	0.5 ± 0.03	-	-
49	*(Z)-*jasmone	1397	nt	0.4 ± 0.07	-	-	-	-
50	methyl eugenol	1407	pp	-	-	0.3 ± 0.02	-	-
51	β-caryophyllene	1419	sh	0.2 ± 0.03	6.7 ± 1.51	1.1 ± 0.15	0.6 ± 0.06	3.1 ± 0.08
**52**	**α-humulene**	**1453**	**sh**	**-**	**1.9 ± 0.39**	**0.3 ± 0.04**	**-**	**3.2 ± 0.10**
53	γ-muurolene	1477	sh	-	0.4 ± 0.08	-	-	-
54	bicyclogermacrene	1496	sh	-	0.3 ± 0.05	-	-	-
55	*trans*-γ-cadinene	1514	sh	-	0.4 ± 0.07	-	-	-
56	δ-cadinene	1524	sh	-	0.9 ± 0.20	-	-	-
57	caryophyllene oxide	1582	os	0.4 ± 0.09	4.3 ± 0.16	0.6 ± 0.10	1.5 ± 0.24	0.8 ± 0.02
58	humulene oxide II	1608	os	0.3 ± 0.07	0.5 ± 0.08	-	-	0.6 ± 0.03
59	caryophylla-4(14),8(15)-dien-5-ol (unidentified isomer)	1633	os	-	0.2 ± 0.06	-	0.3 ± 0.04	-
60	T-cadinol	1641	os	-	0.3 ± 0.03	-	0.2 ± 0.03	-
61	α-bisabolol oxide B	1655	os	-	-	-	0.8 ± 0.06	-
**62**	**14-hydroxy-9-*epi-(E)-*caryophyllene**	**1670**	**os**	**-**	**-**	**-**	**7.1 ± 1.76**	**0.2 ± 0.01**
63	α-bisabolol	1685	os	-	-	-	-	0.4 ± 0.01
64	*trans*-ferruginol	2325	od	0.2 ± 0.04	0.2 ± 0.01	-	-	-
	**Total identified (%)**			**98.6 ± 0.06**	**98.7 ± 0.31**	**99.1 ± 0.16**	**96.6 ± 0.16**	**100 ± 0.03**
				Boule	Gori	Joyce	Vicom	Jord
	Monoterpene hydrocarbons (mh)			50.2 ± 3.77 ^A^	24.0 ± 0.54 ^C^	36.0 ± 0.19 ^B^	14.3 ± 0.46 ^D^	14.2 ± 0.49 ^D^
	Oxygenated monoterpenes (om)			46.9 ± 3.42 ^C^	57.5 ± 2.08 ^B^	60.9 ± 0.04 ^B^	71.9 ± 1.55 ^A^	77.1 ± 0.25 ^A^
	Sesquiterpene hydrocarbons (sh)			0.2 ± 0.03 ^C^	10.7 ± 2.35 ^A^	1.3 ± 0.19 ^C^	0.6 ± 0.06 ^C^	6.3 ± 0.18 ^B^
	Oxygenated sesquiterpenes (os)			0.7 ± 0.16 ^C^	5.3 ± 0.33 ^B^	0.6 ± 0.10 ^C^	9.8 ± 2.12 ^A^	2.0 ± 0.07 ^C^
	Oxygenates diterpenes (od)			0.2 ± 0.04 ^A^	0.2 ± 0.01 ^A^	- ^B^	- ^B^	- ^B^
	Phenylpropanoids (pp)			-	-	0.3 ± 0.02	-	0.4 ± 0.02
	Other non-terpene derivates (nt)			0.4 ± 0.07 ^B^	1.0 ± 0.17 ^A^	- ^C^	- ^C^	- ^C^
	**EO Extraction yield (%*w/w*)**			**0.57 ± 0.02 ^C^**	**1.17 ± 0.16 ^B^**	**0.76 ± 0.04 ^C^**	**2.25 ± 0.15 ^A^**	**0.71 ± 0.04 ^C^**

^1^ Linear retention index on a HP 5-MS capillary column; ^2^ For all chemical classes, and for the extraction yield, different superscript uppercase letters (A–D) indicate statistically significant differences between each sample. The statistical significance of the relative abundances was established by Tukey’s *post-hoc* test, with *p* ≤ 0.05. ^3^ Compounds and values in bold are referred to the compounds evidenced in the statistical analysis.

**Table 2:** The inhibition zones expressed in millimeters resulted from the application of different antibiotics against the selected bacterial strains (S: susceptible; R: resistant; I: intermediate).

Antibiotics						
STRAINS	Tetracycline (30 μg/disc)	Ceftazidime (30 μg/disc)	Rifampicin (30 μg/disc)	Cephalexin (30 μg/disc)	Cefotaxime (30 μg/disc)	Chloramphenicol (30 μg/disc)
*S.* ser. Typhimurium (S176)	18 (S)	19 (S)	15 (R)	21 (S)	25 (S)	21(S)
*Y. enterocolitica* (YU3)	22 (S)	27 (S)	17 (I)	0 (R)	32 (S)	22 (S)
*L. monocytogenes* (L1)	26 (S)	0 (R)	28 (S)	21 (S)	10 (R)	22 (S)
*E. durans* (EU157)	24 (S)	0 (R)	33 (S)	14 (R)	0 (R)	19 (S)
*E. faecium* (EU107)	7 (R)	0 (R)	30 (S)	0 (R)	0 (R)	18 (S)
*E. faecalis* (EU37)	10 (R)	0 (R)	15 (R)	13 (R)	18 (I)	19 (S)

**Table 3:** Antimicrobial activity: Results of the agar diffusion test of the tested EOs at 10% on bacterial strains. Growth inhibition zone expressed in millimeters.

Strain	Boule	Gori	Joyce	Vicom	Jord
*S*. ser. Typhimurium (S176)	7.0 ± 0.0	7.0 ± 0.0	7.0 ± 0.0	8.0 ± 0.0	7.0 ± 0.0
*Y. enterocolitica* (YU3)	8.0 ± 1.0	8.0 ± 0.0	8.0 ± 0.0	8.3 ± 0.6	9.3 ± 0.6
*L. monocytogenes* (L1)	0.0 ± 0.0	7.0 ± 0.0	8.0 ± 0.0	7.7 ± 0.6	7.7 ± 0.6
*E. durans* (EU157)	0.0 ± 0.0	0.0 ± 0.0	0.0 ± 0.0	0.0 ± 0.0	0.0 ± 0.0
*E. faecium* (EU107)	0.0 ± 0.0	0.0 ± 0.0	0.0 ± 0.0	0.0 ± 0.0	0.0 ± 0.0
*E. faecalis* (EU37)	7.0 ± 0.0	0.0 ± 0.0	0.0 ± 0.0	0.0 ± 0.0	7.0 ± 0.0

**Table 4:** MIC values (% *v/v*) of the tested EOs against the selected bacterial strains.

Strain	Boule	Gori	Joyce	Vicom	Jord
*S*. ser. Typhimurium (S176)	>10	>10	2.5	>10	5
*Y. enterocolitica* (YU3)	5	5	1.25	2.5	1.25
*L. monocytogenes* (L1)	10	10	1.25	5	2.5
*E. durans* (EU157)	2.5	5	1.25	1.25	1.25
*E. faecium* (EU107)	5	5	1.25	1.25	1.25
*E. faecalis* (EU37)	>10	10	2.5	5	5

**Table 5:** Antimycotic activity MIC values (% *v*/*v*) of the selected EOs against the yeast strains.

EOs	*C. albicans*	*C. guilliermondii*	*C. tropicalis*	*S. cerevisiae*	*C. parapsilosis*	*C. krusei*
Boule	>10	>10	>10	>10	>10	>10
Gori	>10	>10	>10	>10	>10	>10
Joyce	>10	>10	>10	>10	>10	>10
Vicom	>10	>10	>10	>10	>10	>10
Jord	>10	>10	>10	>10	>10	>10

**Table 6:** Botanical description of the four *Salvia rosmarinus* cultivars (‘Boule’; ‘Gorizia’; ‘Joyce de Baggio’; ‘Vicomte de Noailles’) and *Salvia jordanii*.

Samples	Images	Botanical Description
***Salvia rosmarinus* ‘Boule’**	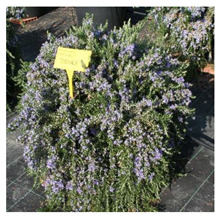	**Voucher N° HMGBH.e/7219.2021.003** Variety with spreading habit. Dense and vigorous vegetation forming a powerful ground cover. Branches: silvery, may fall in long cascades over a wall. Leaves: grey-green, silvery branches. Sky blue flowers. Height: 60 cm. Width: 3 m and up. Hardiness: −12 to −15 °C.
***Salvia rosmarinus* ‘Gorizia’**	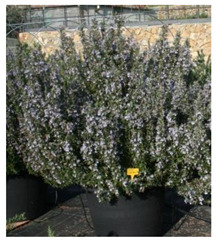	**Voucher N° HMGBH.e/7219.2021.001** Upright variety, vigorous vegetation Leaves: dark green, larger than those of other varieties. Flowers: large, pale blue speckled with purple. Height: till 1.8 m. Width: 1 m. Hardiness: −10 to −12 °C.
***Salvia rosmarinus* ‘Joyce de ’Baggio’**	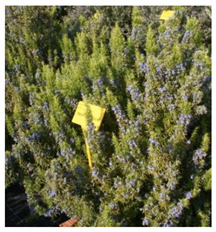	**Voucher N° HMGBH.e/7219.2021.002** Variety with erect shoots with golden foliage in spring. Leaves: very thin and green with yellow edges, becoming green in summer, slightly glutinous, particularly aromatic. Flowers: blue sky. Height: 60–100 cm. Width: 60 to 80 cm. Hardiness: −10 to −12 °C.
***Salvia rosmarinus* ‘Vicomte de Noailles’**	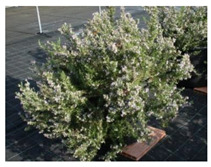	**Voucher N° HMGBH.e/7219.2021.004** Variety with irregular branch habit: some branches are erect, and others are rampant. Leaves: green. Flowers: pink, finely dotted with purple. Height: 60–100 cm. Width: 80 cm. Hardiness: −8 to −10 °C.
***Salvia jordanii*** J.B.Walker	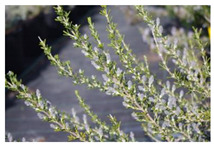	**Voucher N° HMGBH.e/7219.2021.005** Aromatic shrub Branches: grey and procumbent Leaves: 5–15 by 1–2 mm, linear, leathery, with rolled margins, appearing hairless and green profusely hairy on flower stalks. Flowers: green or purplish calyx of 3–4 mm when young, later 5–7 mm; pale blue corolla of 10–12 mm. Height: up to 1.5 m.

## Data Availability

Data is contained within the article.
